# Ultrafast formation of interlayer hot excitons in atomically thin MoS_2_/WS_2_ heterostructures

**DOI:** 10.1038/ncomms12512

**Published:** 2016-08-19

**Authors:** Hailong Chen, Xiewen Wen, Jing Zhang, Tianmin Wu, Yongji Gong, Xiang Zhang, Jiangtan Yuan, Chongyue Yi, Jun Lou, Pulickel M. Ajayan, Wei Zhuang, Guangyu Zhang, Junrong Zheng

**Affiliations:** 1 College of Chemistry and Molecular Engineering, Beijing National Laboratory for Molecular Sciences, Peking University, Beijing 100871, China; 2 Department of Chemistry, Rice University, 6100 Main Street, Houston, Texas 77005-1892, US; 3 Department of Materials Science and NanoEngineering, Rice University, 6100 Main Street, Houston, Texas 77005-1892, US; 4 Beijing National Laboratory for Condensed Matter Physics and Institute of Physics, Chinese Academy of Sciences, Beijing 100190, China; 5 Department of Chemical Physics, University of Science and Technology of China, Hefei 230026, China; 6 State Key Laboratory of Structural Chemistry, Fujian Institute of Research on the Structure of Matter, Chinese Academy of Sciences, Fuzhou 350002, China

## Abstract

Van der Waals heterostructures composed of two-dimensional transition-metal dichalcogenides layers have recently emerged as a new family of materials, with great potential for atomically thin opto-electronic and photovoltaic applications. It is puzzling, however, that the photocurrent is yielded so efficiently in these structures, despite the apparent momentum mismatch between the intralayer/interlayer excitons during the charge transfer, as well as the tightly bound nature of the excitons in 2D geometry. Using the energy-state-resolved ultrafast visible/infrared microspectroscopy, we herein obtain unambiguous experimental evidence of the charge transfer intermediate state with excess energy, during the transition from an intralayer exciton to an interlayer exciton at the interface of a WS_2_/MoS_2_ heterostructure, and free carriers moving across the interface much faster than recombining into the intralayer excitons. The observations therefore explain how the remarkable charge transfer rate and photocurrent generation are achieved even with the aforementioned momentum mismatch and excitonic localization in 2D heterostructures and devices.

## Abstract (web summary)

Van der Waals heterostructures, fabricated via vertical stacking of two-dimensional materials, hold promise for opto-electronic applications. Here, the authors study the exciton-assisted charge transfer mechanisms occurring in a WS_2_/MoS_2_ heterojunction via ultrafast microspectroscopy.

## Graphical abstract

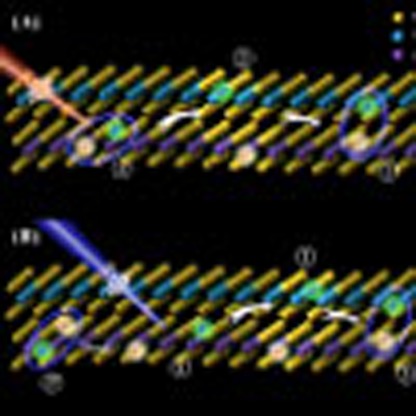

Two-dimensional (2D) materials, including graphene, hexagonal boron nitride, layered transition-metal dichalcogenides (TMDCs), as well as black phosphorus, have demonstrated a wide variety of unique optical, electrical, thermal and mechanical properties^1^,^2^,^3^,^4^,^5^,^6^,^7^,^8^. Atomically thin opto-electronic devices produced by stacking layers of 2D materials^9^,^10^,^11^ to form Van der Waals (VDW) heterostructures have demonstrated fascinating effects, including high mobility, high carrier inhomogeneity^12^ and high on/off ratios in vertical tunnelling transistors^13^,^14^,^15^,^16^,^17^,^18^.

Heterostructures composed of TMDC monolayers are of particular interest for fabricating light-harvesting and photoelectron devices^10^,^14^,^19^. TMDCs are known as the direct bandgap semiconductors with remarkably strong light–matter interactions^7^,^8^,^20^,^21^,^22^,^23^. The efficient separation of photo-excited electrons/holes and generation of across-layer flow of carriers and photocurrent were observed in TMDC heterostructures^24^,^25^,^26^,^27^. The observations further corroborate the promising perspective of the heterostructures as atomically thin opto-electronic devices, at the same time, raise an intriguing question^28^: how can an electron–hole pair, created by light, in these heterostructures undergo an efficient and fast charge separation that leads to photocurrent, despite the momentum mismatch between the layers, as well as the tightly bound nature of excitons in the 2D geometry? The relative lattice orientations of the two TMDC monolayers in a heterostructure are rarely aligned in real or momentum spaces^28^. As a result, electrons transferring across the heterojunction are expected to be accompanied by a large momentum change, and therefore significantly retarded^28^. At the same time, due to the poor screening of Coulomb potential in the 2D geometry, the binding energy of intra/inter layer excitons are 1–2 orders of magnitude higher than that of a typical Mott–Wannier exciton. These tightly bound electron–hole pairs need to overcome a high Coulomb barrier that is much higher than the thermal energy to dissociate and contribute to the photocurrent.

Here, to unravel this fundamentally important puzzle, we carry out ultrafast visible/infrared (IR) microspectroscopy studies on the cross-layer charge transport dynamics in WS_2_/MoS_2_ heterostructure. Different from the visible detection light used in previous works^19^,^27^, the energy of mid-IR detection pulse is well below the excitonic bands, as well as the exciton-binding energies in TMDC monolayers^28^,^29^,^30^. The transient absorption signals are therefore not affected by the tightly bound excitons, but instead sensitive to the photo-excited free carriers, as well as the weakly bound electron/hole pairs. Both species absorb low-energy photons to generate intraband transitions^31^. Our study provides unambiguous experimental evidence showing that interlayer charge transfers are stepwise: the charge transfers result in an intermediate state of electron/hole pair with excess energy before the formation of tightly bound interlayer excitons. The excess energy of the intermediate state (denoted as hot exciton^28^) available allows the sampling of a broader range of momentum space than that determined by the conduction band minimum (CBM) and valence band maximum (VBM) of the stacked heterojunction. Momentum conservation can therefore be overcome. At the same time, the excess energy in the hot intermediates leads to lower-binding energy and a larger electron–hole distance than those of the interlayer excitons. As the result, the intermediates may easily dissociate and contribute to the photocurrent in the presence of the internal/external potentials before relaxing to the interlayer excitons. The results therefore reveal the mechanism how the momentum mismatch and excitonic localization in 2D heterostructures are overcome to reach fast and efficient charge transfers, and photocurrent generation.

## Results

### Charges can efficiently transfer between MoS_2_ and WS_2_

**Figure 1:**
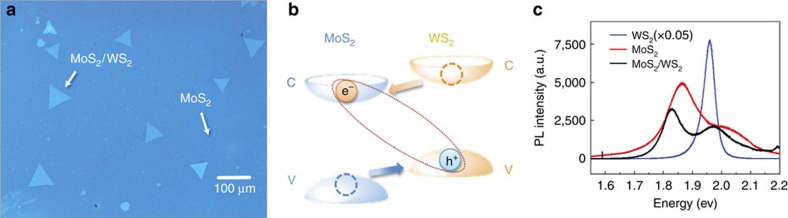
Properties of the MoS_2_/WS_2_ heterostructure. (**a**) Optical microscope image of an as-grown WS_2_/MoS_2_ heterostructure sample. Bright triangle areas correspond to WS_2_ single crystals covered with the MoS_2_ monolayer in the entire image. (**b**) Illustration of the band alignment of a MoS_2_/WS_2_ heterostructure. C, CBM; V, VBM. Optical excitation of the MoS_2_/WS_2_ heterostructure leads to tightly bound interlayer excitons, with electrons (e^−^) in MoS_2_ layer and holes (h^+^) in WS_2_ layer. (**c**) Photoluminescence (PL) spectra of as-grown WS_2_ monolayer, MoS_2_ monolayer and MoS_2_/WS_2_ heterostructure. The strong PL at 1.96 eV of the WS_2_ monolayer and at 1.86 eV of the MoS_2_ monolayer arise from their A-exciton resonances. Photoluminescences in the MoS_2_/WS_2_ heterostructure are quenched, compared with those in the standalone samples, indicating interlayer charge transfers in the heterostructure.

One optical image of the atomically thin MoS_2_/WS_2_ heterostructures is displayed in Fig. 1a. The bright triangles are the monolayer WS_2_ single crystals underneath a monolayer of MoS_2_ that covers the entire image. The samples were epitaxially grown via a two-step chemical vapour deposition method^32^. The well-defined WS_2_ triangular domains with ∼100 μm lateral size were firstly grown on SiO_2_/Si followed by a second time growth of overlaid MoS_2_ layers. The top MoS_2_ layers only have 0° and 60° relative orientations to the bottom WS_2_ layer corresponding to a AA and AB stacking, respectively. By controlling growth conditions, the top MoS_2_ domains coalesced into a continuous monolayer film as illustrated in Fig. 1a. Then, the as-grown WS_2_ single crystals and MoS_2_/WS_2_ VDW heterostructures were transferred onto CaF_2_ substrates for spectroscopic investigations. Raman spectroscopy and atomic force microscopy measurements (Supplementary Figs 1–3) confirm that each component of the hybrid is a monolayer.

The band alignment of the MoS_2_/WS_2_ heterostructure is illustrated in Fig. 1b. The MoS_2_/WS_2_ heterostructure forms a type II heterojunction with the highest VBM and the lowest CBM residing in WS_2_ and MoS_2_ layers, respectively. The offset between two CBMs (VBMs) is 0.27 eV (0.35 eV)^19^,^25^. The optical excitation of the MoS_2_/WS_2_ heterostructure can lead to a tightly bound interlayer exciton with the electron (e^−^) in MoS_2_ and the hole (h^+^) in WS_2_ (refs 19, 25). The efficient charge transfer in the MoS_2_/WS_2_ heterostructure is confirmed by the photoluminescence (PL) measurements. Figure 1c displays the PL spectra of individual monolayer WS_2_, MoS_2_ and as-grown MoS_2_/WS_2_ heterostructure. The sharp peak at 1.96 eV of WS_2_ (blue) and the peak at 1.86 eV of MoS_2_ (red) arise from their A-exciton resonances. In the MoS_2_/WS_2_ heterojunction, the peak of MoS_2_ slightly red shifts, and that of WS_2_ slightly blue shifts, compared with the corresponding peaks of individual monolayers. The energy shifts are caused by the interlayer coupling. The coupling strength is calculated to be ∼0.05 eV. Calculation details are shown in Supplementary Note 1. The PL intensity of WS_2_ in the heterostructure is significantly reduced by >95% compared with that of the monolayer, indicating a very effective charge transfer in the heterojunction since a pair of spatially separated electron and hole in two TMDC layers cannot emit efficiently^19^,^27^.

### Hot excitons form rapidly after the charge transfer

**Figure 2:**
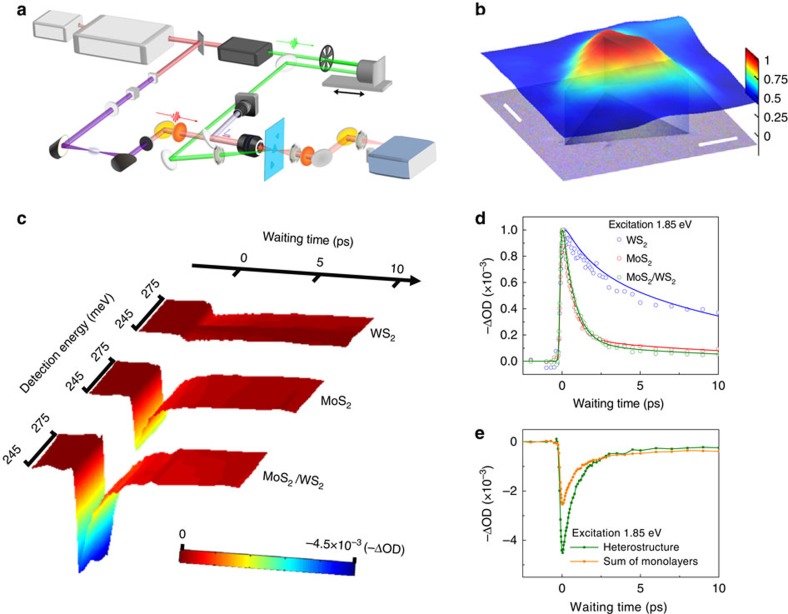
Ultrafast visible/IR microspectroscopy measurements. (**a**) A schematic illustration of ultrafast visible/IR microspectroscopy. (**b**) Image of transient absorption change for a 100 × 100 μm area of MoS_2_/WS_2_ heterostructure sample. The bottom layer of the figure is the optical image of the measured area, where the heterojunction is located at the centre. Scale bar, 20 μm. The top layer represents the transient absorption change 100 fs after the excitation of MoS_2_ with a pulse centred at 1.85 eV. The intensity of signal from the MoS_2_/WS_2_ heterojunction region is about twice of that from MoS_2_-only region. (**c**) Temporal evolutions of the 670 nm (1.85 eV) excitation-induced absorption changes detected at 0.245–0.275 eV of MoS_2_, WS_2_ and the MoS_2_/WS_2_ heterostructure. OD, optical density. (**d**) Normalized plots of (**c**) detected at 0.25 eV (2,000 cm^−1^). Dots are data, and curves are multi-exponential fitting with the consideration of instrument response function (IRF). (**e**) The signal of the MoS_2_/WS_2_ heterostructure and the signal sum of the two individual MoS_2_ and WS_2_ monolayers. The initial intensity of the heterostructure is nearly 100% larger than the signal sum of the two monolayers. All measurements were made at room temperature with incident pump fluence ∼80 μ J cm^−2^. The photo-excited carrier densities is ∼10^12^–10^13^ carriers per cm^−2^.

The real time charge transfer dynamics across the heterojunction were directly monitored with the ultrafast visible/IR microspectroscopy (Fig. 2a). In these experiments, a femtosecond energy-tunable visible pulse excites electrons in the sample from the valence band to the conduction band. The evolution of the photo-excited charge carriers is then monitored by recording the absorption change in the mid-IR region with a femtosecond ultra-broadband mid-IR pulse. In the studies, three sets of experiments were carried out, with the energy values of the visible excitation pulses centred at the A-exciton energy of MoS_2_ (1.85 eV), at the A-exciton energy of WS_2_ (1.95 eV) and at the free carrier energy (3.1 eV), respectively. Different carriers and charge transport dynamical processes were therefore generated in the heterostructure and monitored by the IR detection pulses, providing a comprehensive picture for the across-layer charge transfer dynamics.

Figure 2b displays an image of transient absorption change for a MoS_2_/WS_2_ heterostructure sample. The top layer represents the transient absorption change 100 fs after the excitation of MoS_2_ with a pulse centred at 1.85 eV. The intensity of signal from the MoS_2_/WS_2_ heterojunction region is about twice of that from the MoS_2_-only region. The bottom layer of the figure is the optical image of the measured area, where the heterojunction is located at the centre.

Figure 2c (raw data) and Fig. 2d (normalized data) display the evolutions of the photo-excitation-induced absorption changes in MoS_2_ monolayer (red), WS_2_ monolayer (blue) and the MoS_2_/WS_2_ heterostructure (green) by exciting the samples with a pulse centred at 670 nm (1.85 eV) and detecting at ∼2,000 cm^−1^ (∼0.25 eV). Signals in all samples quickly reach maximum within fs time scales, and then decay in ps time scales. In the WS_2_ monolayer, the rising time is 120 fs, while in MoS_2_ and the heterostructure it is 50 fs. A marked enhancement of the signal maximum is observed (nearly 100% larger than the sum of the two standalone monolayers) in the heterostructure (Fig. 2c,e).

In the experiments, the optical excitation energy is centred at the A-exciton energy (1.85 eV) of MoS_2_ with a width ∼0.1 eV, lower than that (1.95 eV) of WS_2_ (refs 19, 25). In MoS_2_, therefore, electrons are excited to both excitonic band and conduction band because the sharp excitonic band absorption overlaps with the broad conduction band absorption^33^, forming both tightly bound excitons and other less tightly bound electron/hole pairs^31^. More detailed discussions about the carrier excitations are provided in Supplementary Fig. 6. The detection energy of ∼0.25 eV is well below the excitonic-binding energy (∼0.7 eV). Because of this, the excitons similar to a charge neutral-insulating gas^34^,^35^ can hardly absorb the detection IR photons. Consequently, the detection signal is predominantly contributed by the absorption of the weakly bound electron/hole pairs. The recombinations of these electron/hole pairs and their reorganizations to form excitons result in the signal decay. In WS_2_, on the other hand, electrons are excited to the tail of the conduction band (absorption spectrum is in literature^33^ and Supplementary Fig. 6), predominantly forming weakly bound electron/hole pairs, and very few A-excitons are generated. In the heterostructure, both MoS_2_ and WS_2_ are excited, and the signal is mainly contributed from the MoS_2_ excitation (Fig. 2c).

**Figure 3:**
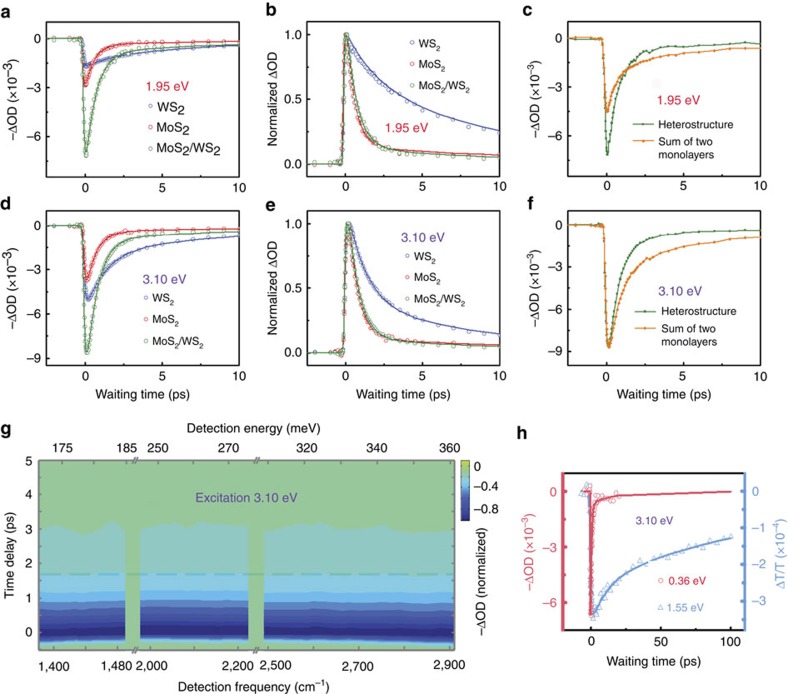
Dynamics following various photon excitations. (**a**) Temporal evolutions of the 630 nm (1.95 eV, fluence 80 μ J cm^−2^) excitation-induced absorption changes detected at 2,000 cm^−1^(0.25 eV) of MoS_2_, WS_2_ and the MoS_2_/WS_2_ heterostructure. Dots are data, and curves are multi-exponential fitting with the consideration of IRF. (**b**) Normalized plots of **a**. (**c**) The signal of the MoS_2_/WS_2_ heterostructure and the simple signal sum of the two individual MoS_2_ and WS_2_ monolayers. (**d**) Temporal evolutions of the 400 nm (3.10 eV, fluence 10 μ J cm^−2^) excitation-induced absorption changes detected at 2,000 cm^−1^ (0.25 eV) of MoS_2_, WS_2_ and the MoS_2_/WS_2_ heterostructure. Dots are data, and curves are multi-exponential fitting with the consideration of IRF. (**e**) Normalized plots of **d**. (**f**) The signal of the MoS_2_/WS_2_ heterostructure and the simple signal sum of the two individual MoS_2_ and WS_2_ monolayers. (**g**) Temporal evolutions of the 400 nm excitation-induced absorption change at frequencies ranging from 1,390 (0.17 eV) to 2,910 cm^−1^ (0.36 eV) for the MoS_2_/WS_2_ heterostructure. The horizontal dashed line represents the time delay at which the signals decay to 20% of initial intensities. (**h**) Temporal evolutions of the 400 nm (3.10 eV) excitation-induced absorption changes detected at 2,910 cm^−1^ (0.36 eV, red) and 800 nm (1.55 eV, blue) of the MoS_2_/WS_2_ heterostructure. Dots are data, and curves are multi-exponential fitting with the consideration of IRF. 1.55 eV is larger than the binding energy of interlayer exciton and the slow signal decay indicates the long excitonic lifetime.

At the interface, charges of both intralayer excitons and weakly bound electron/hole pairs can transfer across the boundary^26^,^27^. Electrons move to MoS_2_ and holes transfer to WS_2_. The crossing-layer charge transfer is supported by the fast decay dynamics (∼0.8 ps) of the heterostructure that is essentially the same as that of MoS_2_, but apparently faster than that (∼2.0 ps) of WS_2_ (Fig. 2d, and also Fig. 3b,e), independent of the relative intensities of the two monolayers. (If charges did not transfer between two layers, the heterostructure decay dynamics would be the weighted average of the two monolayers). Absorption spectrum of MoS_2_ monolayers^33^ reveals that roughly half of the absorbed photons are converted to the energy of the intralayer excitons directly, with the rest going to the weakly bound electron/hole pairs. Detailed discussions of the carrier ratio estimation are in Supplementary Fig. 6. Before charge transfers, the intralayer excitons do not contribute to the detection signals because of their high-binding energy. After the transfer, the intralayer excitons become interlayer charge complexes. The binding energy of the charge complexes is smaller than the detection IR energy, and they absorb the detection IR phonons and enhance the signals, as observed in the experiments. Since the additional IR absorbers generated by the charge transfer of excitons are as many as those generated by the charge transfer of other carriers, the signal is almost doubled. This transfer mechanism is further supported by experiments displayed in Fig. 3d,e, where only free carriers are generated by photo excitation (3.10 eV) and the interlayer charge transfers only change the signal decay dynamics, but not the signal intensity. Detailed discussions will be provided later. Note that this signal enhancement observed might also be caused by the absorption change at the interface, as well as the interlayer coupling. However, the absorption of a heterostructure prepared by vertical heteroepitaxial growth is smaller than the sum of the two standalone monolayers due to interlayer interactions^36^,^37^. Therefore, the absorption change can be excluded as the reason. The increase of signal maximum cannot be caused by the interlayer coupling either, since no signal increase is observed at 3.10 eV excitation (Fig. 3d,f), which should be also affected by interlayer coupling. Multiple reflections between the two monolayers cannot cause the large intensity change either, because of the very small refractive index difference between MoS_2_ and WS_2_ that are shown in Supplementary Fig. 4.

The interlayer charge transfer rate, derived from the signal rising edge of the heterostructures (Fig. 2c,d) is faster than 50 fs. The derivation is shown in Supplementary Note 2. Following the charge transfers, the signal decays very fast. The majority of the signal decays within 800±100 fs, and a small portion decays slower than 13±2 ps. The decays are significantly faster than those of the interlayer excitons detected with visible lights^26^,^27^ and near IR (tens to hundreds of ps; Fig. 3h). The fast decay dynamics, therefore, indicate that the charge transfers do not generate the tightly bound interlayer excitons directly. Instead, the interlayer charge separation immediately produces intermediate hot excitons with binding energy lower than the mid-IR detection energy ∼0.25 eV. The electrons of WS_2_ transfer to the conduction bands above CBM of MoS_2_ and the holes of MoS_2_ move to the valence bands below VBM of WS_2_. The majority of the hot excitons then dissipates the excess energy to the environment and forms the tightly bound interlayer excitons within 800 fs, and a small portion of the hot excitons recombines with 13 ps because of defects or phonon scattering.

The signal enhancement within 50 fs of the heterostructure (Figs 2e and 3c) and the decay dynamics of the heterostructure almost as fast as that of MoS_2_ (Figs 2d and 3b,e) indicate that the formation of interlayer hot excitons (faster than 50 fs) is significantly faster than the formation of intralayer excitons from free carriers or weakly bound charge complexes (600 fs–2.0 ps, Figs 2d and 3b,e).

The charges of excitons in WS_2_ transferring to MoS_2_ lead to a similar phenomena. In the second set of the experiments, the optical excitation energy was centred at 630 nm (1.95 eV). In WS_2_, both the A-excitons (1.95 eV) and weakly bound electron/hole pairs are generated. In MoS_2_, mainly the weakly bound electron/hole pairs are excited, and very few excitons (1.85 eV) are directly generated upon the optical excitation due to their relatively low energy. Similar to the results of experiments with 1.85 eV excitation, the maximum signal intensity of the heterostructure is apparently larger than the signal sum of the two standalone monolayers as displayed in Fig. 3a,c. As discussed above, the signal enhancement is also caused by the formation of interlayer hot exciton intermediates after the electron transfer of the A-excitons of WS_2_ to MoS_2_. The signal dynamics of the heterostructure are identical to those excited by the 1.85 eV photons, despite the relative signal intensity in each monolayer is different under the two excitations.

### Formation of intralayer excitons is relatively slow

When free carriers are created and move across the heterojunction afterwards, no signal enhancement is observed. In the third set of experiments, the excitation pulse is tuned to 400 nm (3.10 eV). The photon energy is much higher than the bandgaps of MoS_2_ (2.39 eV) and WS_2_ (2.31 eV)^19^,^25^. Therefore, free carriers instead of excitons are generated^38^. In the standalone monolayer samples, the free carriers relax to form excitons, contributing to the fast signal decays^16^ (600±100 fs in MoS_2_ monolayer and 1.3±0.2 ps in WS_2_ monolayer) observed in Fig. 3d,e. The slow portion of the signal decays (∼10 ps) is attributed to the recombination of the free carriers through other ways, for example, the carrier-phonon scattering or defect assisted electron–hole recombination^34^. In the heterostructure, both MoS_2_ and WS_2_ are excited by the 3.10 eV light. More carriers are generated in the WS_2_ layer than in MoS_2_ (Fig. 3d). Similar to the observations with excitations at lower energy, the free carriers rapidly transfer across the interlayer boundary, resulting in a much faster signal decay of the heterostructure compared with that of WS_2_. The results clearly demonstrate that the interlayer charge transfers occur before the formation of intralayer excitons. After crossing the interlayer, the free carriers still remain free and able to absorb the mid-IR photons. The population of photo-induced free carriers is unchanged, and the absorption coefficients of carriers are almost unchanged because of the similar environment in MoS_2_ and WS_2_ monolayers, leading to that the interlayer charge transfers following the 3.10 eV excitation do not enhance the signal intensity (Fig. 3f). This is different from the observations with the excitation energy on resonance with the A-excitons (Figs 2e and 3c), where interlayer charge transfers of excitons produce extra mid-IR absorbers, as discussed above.

The observed fast signal decay in the MoS_2_/WS_2_ heterostructure is repeatable in different areas of the sample with different incident excitation fluences (from 5 to 20 μJcm^−2^). As the fluence increases, the excitation-induced absorption change increases with the same proportion and has not reached the nonlinear regime until the incident fluence reaches 20 μJcm^−2^ (Supplementary Fig. 5). Under the excitation conditions, it is possible that biexcitons or other multi-particle species can be generated^39^. These multi-particle species can evolve into exciton pairs with excess energy in a fission-type process. However, the binding energy (∼50 meV)^39^ of these species is lower than the mid-IR-probe photon energy, and they contribute to the experimental signals in a way similar to free carriers that is not affected by interlayer charge transfer. Therefore, the generations of these multi-particle species do not affect the conclusion that the charge transfer of intralayer excitons results in interlayer hot excitons. More discussions about possible high-photoexcitation-density effects on the dynamics are provided in Supplementary Fig. 5. Figure 3g also demonstrates that the photo-induced dynamics in the heterostructure remain constant in the detection energy range from 1,390 (0.17 eV) to 2,910 cm^−1^ (0.36 eV), indicating that the binding energy of the interlayer hot exciton intermediate is <0.17 eV and that of the interlayer excitons is >0.36 eV.

In the above experiments, signals following the interlayer charge transfers decay significantly faster than that of the interlayer excitons observed with visible^19^ and near-IR detections (1.55 eV; Fig. 3h). As discussed above, experiments in this work detect free carriers and intermediate hot excitons with binding energy lower than the detection mid-IR photon energy. Charge complexes, for example, A-excitons, with higher binding energy do not contribute to the experimental signals. The binding energy of interlayer exciton in the MoS_2_/WS_2_ heterostructure has not been previously experimentally determined. Our experiments show that the 1.55 eV near-IR photon can excite the interlayer excitons (Fig. 3h), but the 0.36 eV mid-IR photon cannot (Fig. 3g), indicating that the binding energy of the interlayer excitons resides between 0.36 and 1.55 eV. Our calculations based on a model^28^ also suggest that the interlayer binding energy is 0.4–0.6 eV (Supplementary Fig. 7; Supplementary Note 3), slightly lower than that (0.5–0.7 eV) of a single layer^28^,^29^,^30^.

### The charge transfers include two major steps

**Figure 4:**
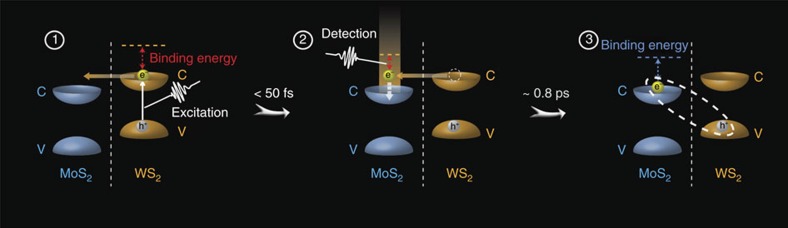
Charge separation processes in the heterostructure. After the excitation of WS_2_ (MoS_2_) monolayer, the electron (hole) transfers from the CBM (VBM) of WS_2_ (MoS_2_) monolayer to the conduction (valence) bands just above (below) CBM (VBM) of the MoS_2_ (WS_2_) monolayer within 50 fs, generating a hot interlayer exciton with a relatively long distance. The hot exciton then reorganizes and dissipates the excess energy to form the tightly bound interlayer exciton within 800 fs.

**Figure 5:**
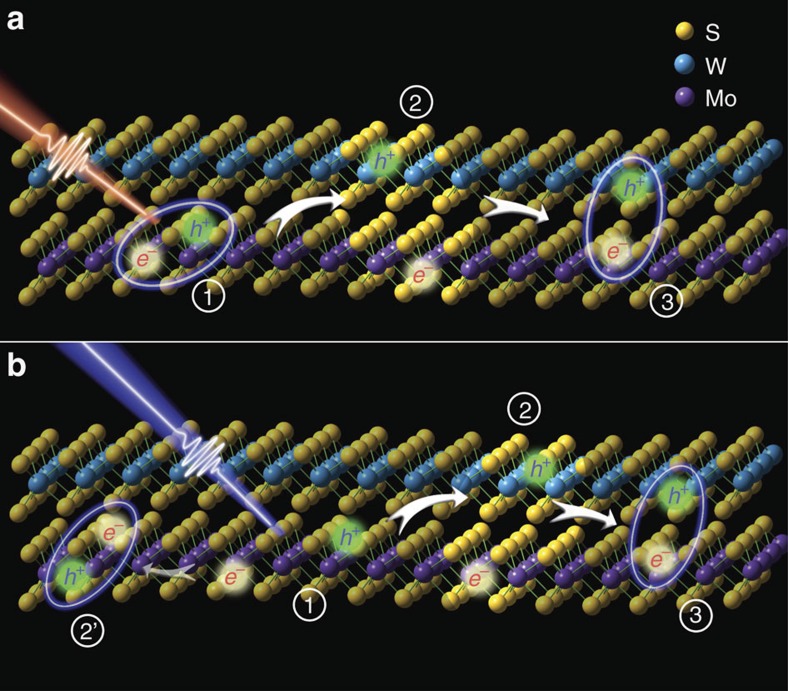
Illustration of the light-induced charge separation and the formation of interlayer exciton in a WS_2_/MoS_2_ heterostructure. After photoexcitation, electron/hole pairs, (**a**) excitons and (**b**) other carriers, are generated in each individual layer (using MoS_2_ as an example), and then holes transfer to WS_2_ and electrons transfer to MoS_2_ to form the intermediate states—interlayer hot excitons, and the hot excitons finally relax to generate the tightly bound interlayer excitons. In **b**, the formation of interlayer intermediate is significantly faster than the formation of intralayer exciton .

Our measurements herein suggest that the interlayer charge transfer process in the MoS_2_/WS_2_ heterostructure includes two major steps: the immediate formation of interlayer hot excitons following the ∼50 fs interlayer charge separation, and then the relaxation of hot excitons to generate tightly bound excitons within ∼800 fs. The process is schematically illustrated in Figs 4 and 5. The experimental observations support one of the two theories (the hot exciton theory) recently proposed^28^ to explain why the charge transfer dynamics in 2D heterostructures are little dependent on the momentum mismatch of intra- and inter-layer excitons: the momentum conservation during the charge transfer can be achieved by the formation of a hot interlayer exciton that employs the excess energy through the transfer. Specifically, after photo excitation, charge transfers don't form interlayer excitons directly. Instead, the electron (hole) transfers from WS_2_ (MoS_2_) to the conduction (valence) bands above (below) CBM (VBM) of the MoS_2_ (WS_2_), generating a hot interlayer exciton. The hot exciton has a relatively long distance between the electron and hole, and therefore is only weakly bound. The hot exciton then reorganizes and dissipates the excess energy to form the tightly bound interlayer exciton.

## Discussion

The experiments herein demonstrate that the ultrafast charge transfer between the WS_2_ and MoS_2_ monolayers results in the formation of interlayer hot excitons. The existence of hot excitons explain why the interlayer charge transfer is independent on the apparent momentum mismatch between the intralayer/interlayer excitons, and also answers why an electron–hole pair can overcome the large Coulomb potential to generate photocurrent in ultrathin photovoltaic cells with MoS_2_/WSe_2_ or MoS_2_/WS_2_ as the active p–n junction and conventional metal or graphene as contacting electrodes^24^,^36^. The excess energy of the hot interlayer exciton can help the electron/hole pair easily dissociate in the built-in potential before they turn into the tightly bound interlayer excitons^28^. That carriers other than excitons created in the heterostructure move across the interface with a much higher rate than recombining into the intralayer excitons also favours the generation of photocurrent. The semi-free character of the hot interlayer excitons can greatly facilitate the charge separation and photocurrent generation processes, which is critical for many applications of 2D heterostructures in optoelectronics and photovoltaics.

## Methods

### MoS_2_/WS_2_ heterostructures growth

Our heterostructure growth follows a two-step process: the WS_2_ domains were firstly grown on SiO_2_ substrates followed by second growth of MoS_2_ on the top of WS_2_. SiO_2_ substrate was pre-cleaned with acetone and isopropanol, followed by treated in piranha solution (H_2_SO_4_:H_2_O_2_—3:1) for 2 h. The triangular WS_2_ domains were firstly grown on SiO_2_ (300 nm)/p++Si substrates. Growth process was done in three-zone chemical vapour deposition system with 1-inch quartz tube using WO_3_ (Alfa Aesar 99.999%), MoO_3_ (Alfa Aesar 99.999%) and S (Alfa Aesar 99.9%) powders as the precursor. For MoS_2_ growth, typical temperatures for three zones are 115 °C, 560 °C and 800 °C, respectively. All of three temperature zones were heated to preset values at a rate of 25 °C min^−1^. Sulfur and MoO_3_ powders were separately loaded in two 10 mm-radius mini- quartz tubes, which enable a stable evaporation of S and MoO_3_ sources by preventing any cross-contaminations during the growth. All temperature zones were kept stable for 20 min before growth. The precursor powders were pre-placed right outside of the furnace and rapidly loaded from outside into each zone to start the growth. During the growth process, argon was used as a carrying gas at a flow rate of 130 sccm, and the vacuum pressure was kept at 0.7 torr.

### Heterostructures transfer

The as-grown MoS_2_/WS_2_ VDW heterostructures are transferred onto CaF_2_ substrate for transmission mode laser experiments. The process is: at first, a thin layer of poly(methyl methacrylate) (PMMA; PMMA-B4 4%wt) is spin-coated onto the sample on SiO_2_ substrate twice at the speed of 4,000 r.p.m., then the sample is carefully placed in the buffer HF (1:5) and let it float. After 20 h, the PMMA sheets is separated from the SiO_2_ substrate and fished with glass plate, move to deionized-water for rinsing a few times, then pre-cleaned CaF_2_ windows are used to fish the PMMA sheets. The CaF_2_ windows with PMMA and samples stay in room temperature and vacuum for 24 h, finally acetone is used to remove the PMMA, the MoS_2_/WS_2_ VDW heterostructures stayed on the CaF_2_ substrate and confirm by optical microscope.

### Raman and PL measurements

Raman and PL spectroscopy were carried out using a Horiba Jobin Yvon LabRAM HR-Evolution Raman microscope. The excitation light is a 532 nm laser, with an estimated laser spot size of 1 μm and the laser power of 1 mW.

### Ultrafast visible/IR microspectroscopy

The experimental set-up of the ultrafast visible/IF microspectroscopy is illustrated in Fig. 2a and Supplementary Fig. 4. In brief, the output of a femtosecond amplifier laser system (at a repetition rate of 1 kHz, 1.6 mJ energy per pulse, 800 nm central wavelength and a pulse duration of ∼50 fs, Uptek Solutions Inc.) was split into two parts. One was used to pump a home-built nonlinear optical parametric amplifier to generate visible laser pulses with tunable wavelengths, and the other was directed to generate an ultra-broadband super-continuum pulse that covers almost the whole mid-IR region^40^,^41^. In ultrafast experiments, the visible pulse is the pump light with the central wavelength and excitation power adjusted based on need. The interaction spot on samples varies from 120 to 250 μm. The mid-IR super-continuum pulse acts as the probe light, which was focused at the sample by a reflective objective lens (× 15/0.28 numerical aperture, Edmund Optics Inc.) to reduce the spot size to the level of sample area (<40 μm). A 300-megapixel microscope digital camera was used to align the pump/probe beam to proper sample area. The probe light was detected by a liquid-nitrogen-cooled mercury–cadmium–telluride array detector after frequency resolved by a spectrograph with a resolution of 1–3 cm^−1^ that is dependent on the central frequency. The time delay between the pump light and probe light was controlled by a motorized delay stage.

### Data availability

The data that support the findings of this study are available from the corresponding author upon request.

## Additional information

**How to cite this article**: Chen, H. *et al*. Ultrafast formation of interlayer hot excitons in atomically thin MoS_2_/WS_2_ heterostructures. *Nat. Commun.* 7:12512 doi: 10.1038/ncomms12512 (2016).

## Supplementary Material

Supplementary Information Supplementary Figures 1-7, Supplementary Notes 1-3, Supplementary References
