# Supplementary material for: Ultrafast formation of interlayer hot excitons in atomically thin MoS2/WS2 heterostructures
Source: Nat Commun. 2016 Aug 19;7:12512. doi: 10.1038/ncomms12512 (PMC4992179; doi:10.1038/ncomms12512)
Supplement: Supplementary Information — Supplementary Figures 1-7, Supplementary Notes 1-3, Supplementary References [file ncomms12512-s1.pdf]

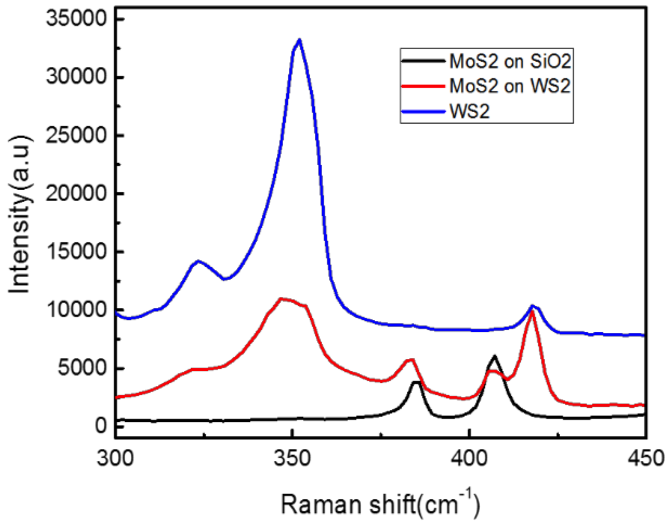

Supplementary Figure 1: Comparing Raman spectra of WS_2/MoS_2 VDW heterostructures with corresponding standalone MoS_2 and WS_2 monolayers. The MoS_2 monolayer has Raman peaks at 407cm^{-1} (E mode) and 385cm^{-1} (A_1 mode), and the WS_2 monolayer has peaks at 352cm^{-1} (E mode) and 418cm^{-1} (A_1 mode). All of these peaks match the intrinsic Raman modes of MoS_2 and WS_2[1, 2]. The Raman spectrum of MoS_2/WS_2 VDW heterostructures (red line) appears to contain all four peaks of Raman modes from two standalone monolayers, indicating the successful fabrication of MoS_2/WS_2 VDW heterostructures.

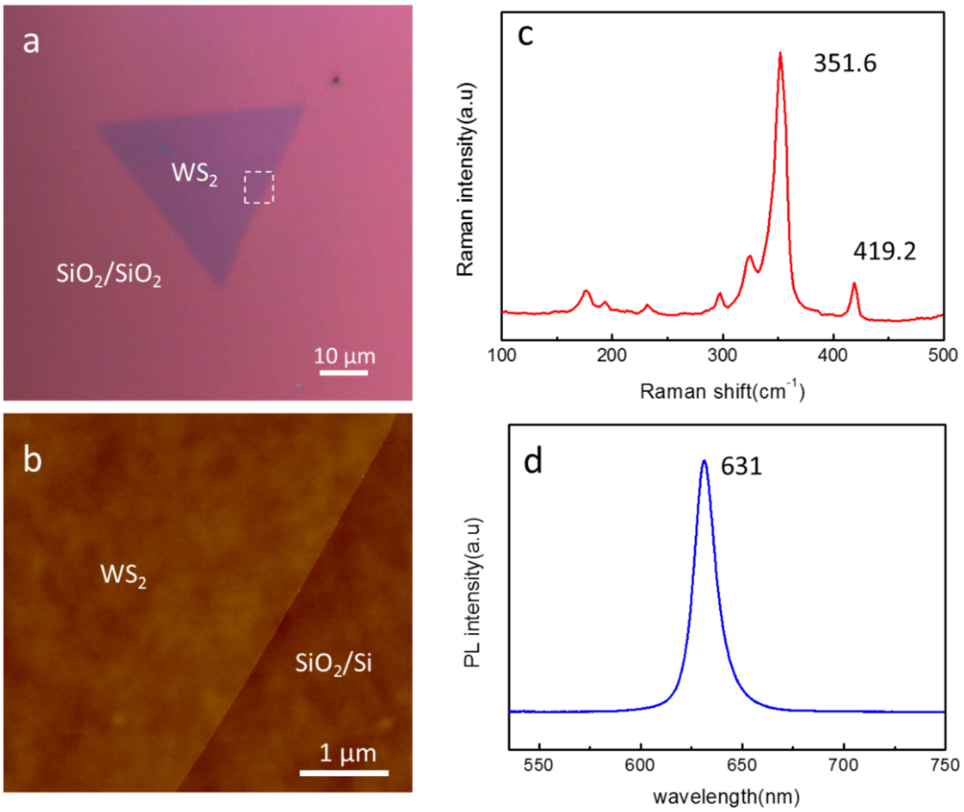

Supplementary Figure 2: Characterization of triangular monolayer WS_2 domain on SiO_2/Si. (a) Optical image of monolayer WS_2 grown on SiO_2/Si. (b) AFM image of as-grown WS_2 on SiO_2/Si substrate from white dashed square in figure 1a. (c)-(d) Typical Raman and PL spectra for monolayer WS_2.

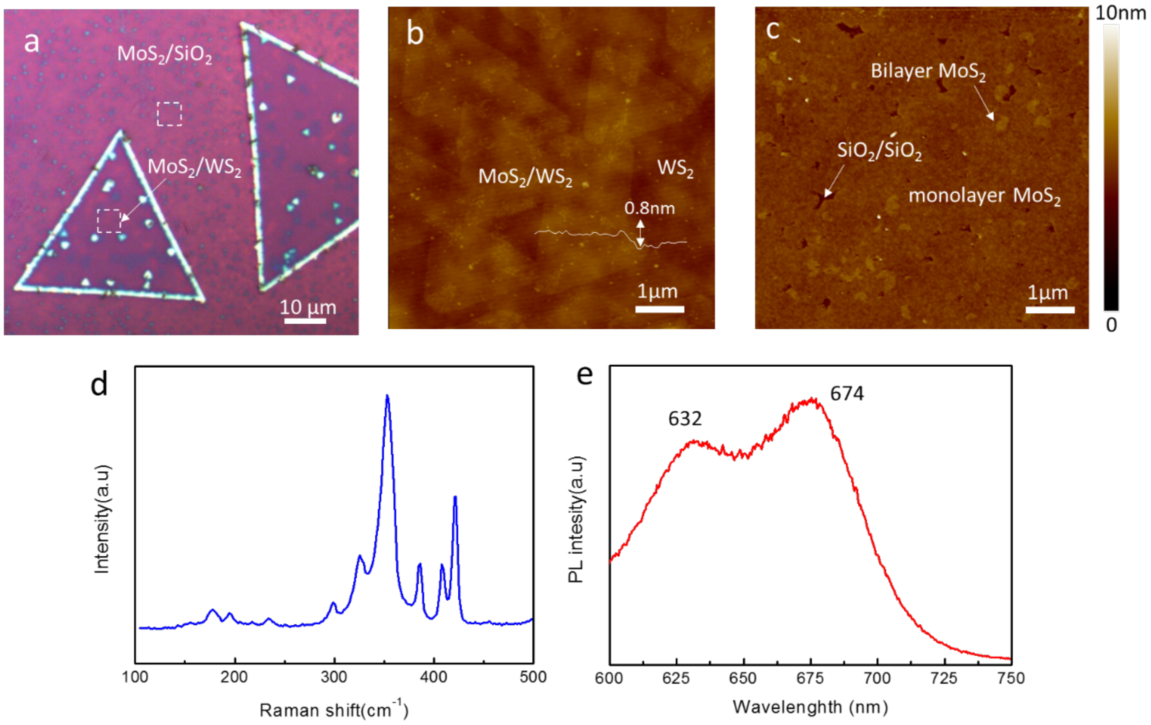

Supplementary Figure 3: AFM, Raman and photoluminescence measurements of the heterostructure. (a) Optical image of WS_2/MoS_2 heterostructures. (b)-(c) AFM images of as-grown WS_2/MoS_2 and monolayer MoS_2 on SiO_2/Si substrate from white dashed square in figure 3a. (d)-(e) Typical Raman and PL Spectra for as-grown MoS_2/WS_2 heterostructure. Both AA and AB stacking were used in the measurements. However, we cannot distinguish them under our experimental condition. About 4-5 flakes were measured, and all of them gave the similar results.

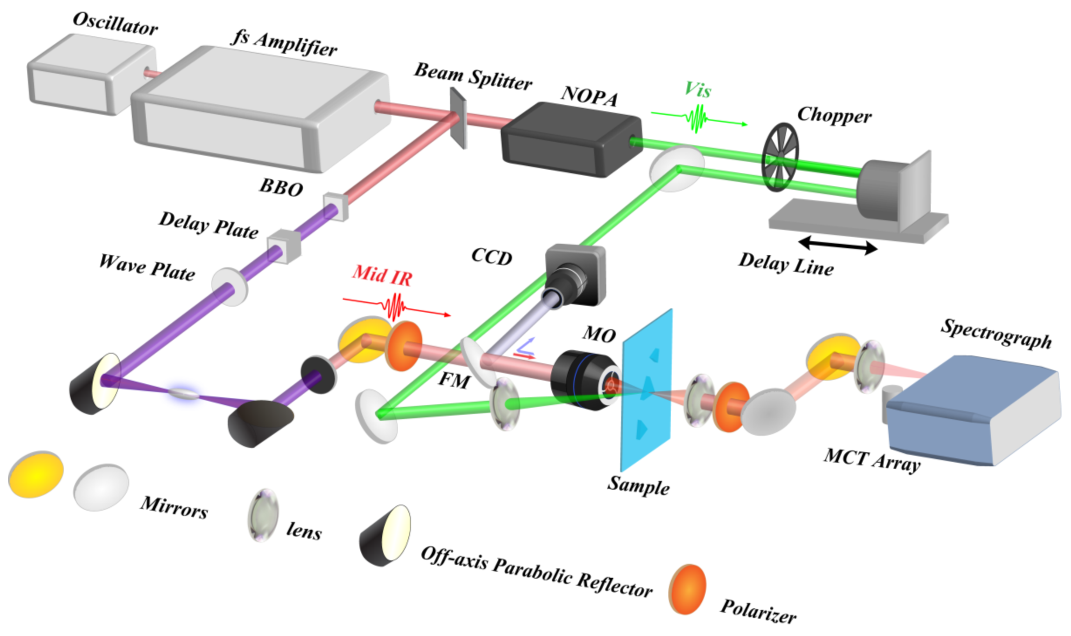

Supplementary Figure 4: Optical setup of ultrafast visible/infrared microspectroscopy. NOPA, home-built nonlinear optical parametric amplifier; MDC, microscope digital camera; MO, reflective microscope objective (15X/0.28NA, Edmund Optics Inc.); FM, flip mirror. The ultra-broadband super-continuum mid-IR pulse is generated by focusing the 800 nm fundament light and 400 nm second harmonic on air[3, 4], with a pulse duration around 110 fs in the frequency range from <20 to >3500 cm^{-1} at 1 kHz. In brief, the 800 nm beam from the fs amplifier is frequency-doubled by passing through a Type-I BBO crystal (150 μm thick, cut at 29.2°) to generate a 400 nm pulse. The wave plate is used to alter relative polarizations of the 800 and 400 nm pulses, and the BBO delay plate (2 mm thick, cut at 55°) is inserted to compensate the temporal walkoff between 800 nm and 400 nm beams. The flip mirror is used to direct the reflected light from the sample into a 300-megapixel microscope digital camera, which allows us to selectively detect different areas of the sample.

The samples were transferred onto CaF_2 substrates for spectroscopic investigations. On one hand, the surface flatness of CaF_2 substrates are far worse than those of Si/SiO_2 substrate. As a result, the interference from multiple reflects on the interface between the sample and the substrate is very weak. This is one reason for the

relatively low contrast ratio of our measured samples under the microscope. On the other hand, the reflectance on the interface between two monolayers can be estimated. By using the dielectric functions of MoS_2 and WS_2 monolayers (Physical Review B 90, 205422 (2014)), the refractive indexes are about 5.1 and 4.5 at 1.85 eV, respectively. According to the Fresnel equations and assuming vertical incidence, the reflectance is $[(5.1-4.5)/(5.1+4.5)]^2$=0.4%, which is very small. Therefore, the interference from multiple reflects on the interface between two monolayers is negligible.

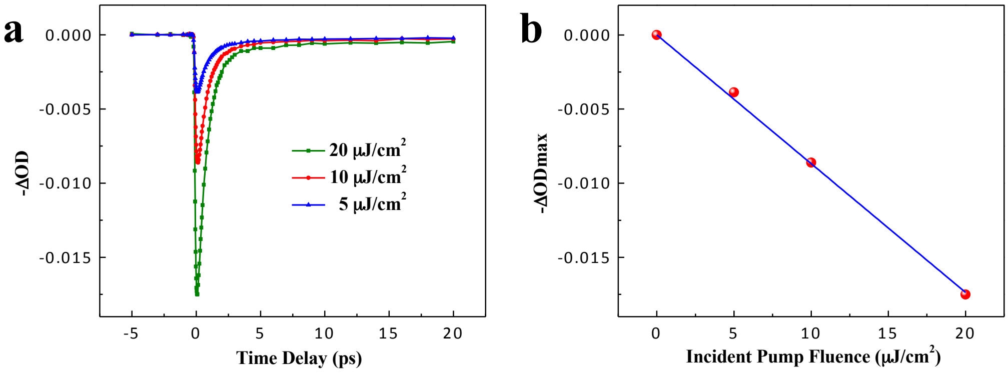

Supplementary Figure 5: Incident pump fluence dependent excitation-induced absorption signals. Excitation-induced absorption change of MoS_2/WS_2 heterostructures after the excitation at 400 nm (3.1 eV). a. Temporal evolution of the excitation-induced absorption change at 2000 cm^{-1} (0.25 eV) under different incident pump fluences. b. The maximum excitation-induced signal (the points) as a function of incident pump fluence, which can be well fitted with a linear function (the line). The incident pump fluence used for 3.1 eV excitation is 10 μJcm^{-2}, located in the linear signal region. Considering the much smaller absorption coefficient at lower energy regions, the incident pump fluence used for 1.85 eV or 1.95 eV excitation is 80 μJcm^{-2}, with photo-excited carrier densities of the same magnitude as the 3.1eV excitation. In the nonlinear region with higher fluences, an abundance of photo-excited carriers will be generated, leading to a significant absorption of the detection light. On one hand, the large change of the absorption usually results in a non-linear relationship between the signal intensity and the population of detected carriers. On the other hand, the high density of photo-excited carriers can also cause other kinds of relaxation pathways via multi-particle interactions, leading to different decay time constants.

Under the excitation conditions, it is possible that biexcitons or other multi-particle species can be generated[5]. However, from our experiments we don't know whether biexcitons are generated in our experiments or not, because their binding energy (~50 meV) is lower than our Mid IR probe photon energy. In terms of detection signals in our experiments, they aren't different from the free carriers or

those weakly bound charge complexes. All have binding energy lower than the probe energy, and all contribute to the detected signal. Even if multi-particle species (e.g. biexcitons) exist after the excitation, it cannot change our conclusions of the hot exciton formation. Because no matter in the standalone layers or the heterostructure, multi-particle excitons are generated under such an excitation if such a generation does occur. These multi-particle excitons immediately contribute to our experimental signal because their binding energy (about 50 meV) is lower than the infrared detection photon energy. Even if these excitons evolve into exciton pairs with excess energy through the fission type, they don't contribute extra signal because the excess energy still makes them experimentally undistinguishable from those weakly bound species. Our conclusion of hot excitons comes from the fact that the initial signal (50 fs) of the heterostructure is much larger than the sum of the two standalone mono layers. There is only one possibility responsible for this observation: some species that originally don’t contribute to the experimental signal inside one layer must evolve into species that contribute to the signal in the heterostructure. The only species upon excitation that originally doesn’t contribute to the signal is the tightly bound intralayer exciton because their binding energy is higher than the detection photon energy. The intralayer exciton must evolve into species with low binding energy in order to contribute the extra signal observed. The species with low binding energy can be interlayer hot exciton, intralayer exciton pairs with excess energy, or interlayer exciton pairs with excess energy. The probabilities of forming intralayer and interlayer exciton pairs with excess energy are similar and therefore the signal of the heterostructure should not be much larger than the sum of the two mono layers if only the exciton pairs are generated. The extra signal in the heterostructure must come from some process that cannot occur within a mono layer. That is, the formation of interlayer hot exciton.

Specifically, if the biexcitons with low binding energy (~50 meV) are formed in MoS_2 or WS_2 monolayers under our excitation conditions, it does contribute to the transient absorption signals. However, after the charge transfer, these photo-excited carriers can either form tightly bound interlayer excitons or form some kind of hot

interlayer excitons with relatively low binding energy. As a result, the signal intensity can only be decreased or unchanged, assuming that the absorption coefficient of one biexciton is twice of that of one free carrier. Therefore, the observed enhancement of the signal (Fig.2E and Fig.3C) can only be contributed by the conversion of photo-excited intralayer excitons (some of these excitions could be the relaxation results of the biexcitons) to the interlayer charge complexes with relatively low binding energy. If the biexcitons don't transfer to the other layer, they will simply relax and cause the signal to decay. Either case cannot increase the signal intensity, and therefore doesn't affect our conclusion that hot interlayer excitons must exist for interlayer charge transfer of intralayer excitons.

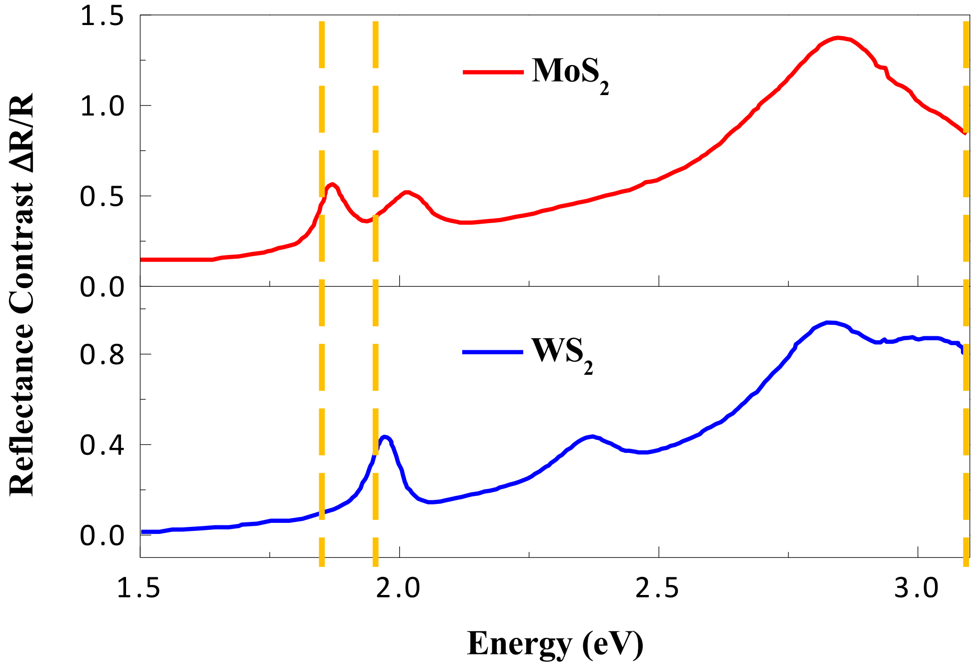

Supplementary Figure 6: Absorption Spectra of WS_2, and MoS_2 from literature[6]. It can be seen from the absorption spectra that, there is an absorption of monolayer WS_2 at 1.85 eV. As a result, the electrons can still be excited to the tail of the conduction band with a pump photon energy of 1.85 eV, predominantly forming weakly-bound electron/hole pairs, leading to the small signal in Fig. 2C. As shown in the figure, the absorption of MoS_2 monolayer at 1.85 eV is composed of two components. The sharp speak around 1.85 eV presents the absorption from the A-exciton, and the background spectrum is contributed by the absorption from the tail of conduction band. The ratio of two absorption is approximate to 1:1. As a result, after the excitation of MoS_2 monolayer with 1.85 eV light, electrons are excited to both excitonic band and conduction band, forming both tightly-bound excitons and other less tightly-bound electron/hole pairs, with approximately equal probability. Considering the absorption coefficients of these two kinds of electron/hole pairs after charge transfer may be different, the intensity ratio of corresponding transient absorption signals is not exactly the same as the absorption ratio of two photo-excited species in MoS_2 monolayer.

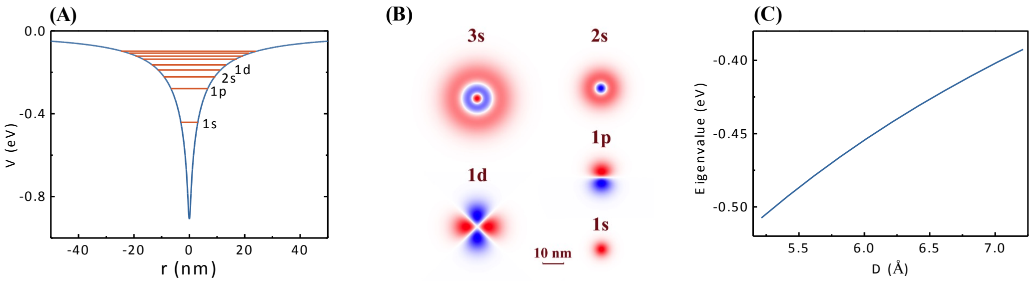

Supplementary Figure 7: Calculations of binding energy of interlayer exciton. (a) A few eigenvalues on the Model Coulomb potential as a function of in-plane radius for an electron-hole pair across the MoS_2/WS_2 van der Waals interface. (b) Wave functions of five CT excitons (red/blue: positive/negative.) (C) Slab distance dependent exciton binding energy.

Supplementary Note 1: The coupling strength between two layers in MoS_2/WS_2 heterojunctions

As displayed in Figure 1C, MoS_2 and WS_2 monolayers show strong photoluminescence at their respective A-exciton resonances:1.86 eV and 1.96 eV. In the MoS_2/WS_2 heterojunction, the peak of MoS_2 slightly red shifts to 1.83 eV, and that of WS2 slightly blue shifts to 1.97 eV. The frequency shifts can be attributed to the weak coupling between the two layers, and the coupling strength γ can be calculated by using a coupled two-system model. From two standalone monolayers, we have the energies of A-exciton resonances, i.e., E_1=1.86 eV for MoS_2 and E_2=1.96 eV for WS_2. In the MoS_2/WS_2 heterojunction, with the consideration of interlayer coupling, the energies of observed A-exciton resonances in heterojunction could be obtained by finding the solutions λ of the following secular determinant:

$$\begin{vmatrix} E_1 - \lambda & \gamma \\ \gamma & E_2 - \lambda \end{vmatrix} = 0. \qquad \text{(Supplementary Equation 1)}$$

By replacing λ with 1.83 eV and 1.97 eV, γ can be calculated as 0.06 eV and 0.03 eV, respectively. As a result, the coupling strength could be estimated by choosing the average value ~0.05 eV, which is much smaller than the energy of A-exciton resonance in each standalone monolayer.

Supplementary Note 2: Multi-exponential fitting of waiting time dependent excitation-induced absorption signals

All waiting time dependent excitation-induced absorption signals in this work can be well fitted by an multi-exponential function convoluted with instrument response function (IRF):

$$-\Delta OD(t) = -[A_1 \exp(-t/\tau_1) + A_2 \exp(-t/\tau_2) + A_3 \exp(-t/\tau_3)] \otimes i(t), \quad \text{(Supplementary Equation 2)}$$

where $\otimes$ represents the convolution, $-\Delta OD(t)$ is the intensity of excitation-induced absorption change at waiting time t (the time delay between the pump light and probe light), τ_1-τ_3 are time constansts, and coefficients A_1 and A_i (i = 2, 3) are confined to be negtive and positve representing the rise and decay of the signal, respectively. $i(t)$ is a Gaussian function of IRF:

$$i(t) = \frac{2\sqrt{\ln 2}}{\Delta\sqrt{\pi}} \exp\{-\ln 2 \cdot [2(t-t_0)/\Delta]^2\} \quad \text{(Supplementary Equation 3)}$$

where t_0 represents the zero point, and Δ is full width at half maximum (FWHM) of the IRF, which is about 150 fs in current work.

The fitting time constants are listed below:

In Figure 3B, τ_1=0.12±0.06 ps, τ_2=1.7±0.6 ps, and τ_3=13±2 ps for WS_2; τ_1 =0.05±0.01 ps, τ_2=0.63±0.02 ps, and τ_3=13±2 ps for MoS_2; τ_1=0.05±0.01 ps, τ_2 =0.79±0.03 ps, and τ_3=11±2 ps for MoS_2/WS_2 heterostructure.

In Figure 2D, τ_1=0.03±0.01 ps, τ_2=2.0±0.3 ps, and τ_3=10±1 ps for WS_2; τ_1 =0.08±0.01 ps, τ_2=0.56±0.03 ps, and τ_3=14±3 ps for MoS_2; τ_1=0.07±0.01 ps, τ_2 =0.68±0.04 ps, and τ_3=11±3 ps for MoS_2/WS_2 heterostructure.

In Figure 3E, τ_1=0.10±0.01 ps, τ_2=1.3±0.1 ps, and τ_3=10±1 ps for WS_2; τ_1

=0.09±0.01 ps, τ_2 =0.63±0.02 ps, and τ_3 =14±2 ps for MoS_2; τ_1 =0.12±0.01 ps, τ_2 =0.71±0.02 ps, and τ_3 =12±1 ps for MoS_2/WS_2 heterostructure.

Supplementary Note 3: Calculation methods

First-principles calculations of monolayer MoS_2 and WS_2, MoS_2/WS_2 2D heterostructures system are conducted using DFT methods implemented in the Vienna ab initio simulation package (VASP). (cite: Kresse G., Furthmüller J. Phys. Rev. B 1996, 54, 11169-11186.) The projector-augmented-wave (PAW) pseudopotentials and the generalized gradient approximation of Perdew, Burke, and Ernzerhof (PBE) for exchange-correlation functional are adopted in our simulations. (cite: Perdew J. P., Burke K., Ernzerhof M. Phys. Rev. Lett. 1996, 77, 3865-3868.) We used a 23×23×4 k-meshes for Brillouin zone (BZ) sampling in the Monkhorst-Pack scheme. (cite: H. J. Monkhorst and J. D. Pack, Phys. Rev. B: Condens. Matter Mater. Phys., 1976, 13, 5188–5192.) Furthermore, van der Waals interactions are taken into account by using the semiempirical DFT-D3 method by Grimme. (cite: Grimme, S.; Antony, J.; Ehrlich, S.; Krieg, H. J. Chem. Phys. 2010, 132, 154104.) The energy cutoff is set to 500 eV for a plane-wave expansion. All atomic coordinates are relaxed until the atomic forces were below 0.001 eV/Å, enforcing a total energy convergence criterion of 1×10^{-7} eV. To avoid spurious interactions with replicas, a vacuum slab larger than 15 Å is added in the z direction.

The optimized lattice parameters of monolayer MoS_2 and WS_2 are a = 3.164 Å and a = 3.169 Å, which is consistent with experimental value (a = 3.15 Å for MoS_2 and a = 3.153 Å for WS_2). (cite: N. Wakabayashi, H. G. Smith, and R. M. Nicklow, Phys. Rev. B 12, 659 (1975). and W. J. Schutte, J. L. de Boer, and F. Jellinek, J. Solid State Chem. 70, 207 (1987).) Aware of their lattice parameters almost the same, only 0.16% errors, the lattice mismatch between MoS_2 and WS_2 could be ignored safely. Then, the optimized lattice parameters of MoS2/WS2 heterojunction is a = 3.166 Å, and the thickness between the two layers is 6.213 Å. Furthermore, the in-plane dielectric constant of MoS_2/WS_2 heterostructures is $\bar{\varepsilon}$ = 10.72, which is average by the calculated in-plane dielectric constant ε_{xx} = 15.98 and 5.47 for monolayer MoS_2 and WS_2, respectively. To obtain the potential experienced by an electron at (ρ, z) due to the presence of a hole at (0, z_0), we using a field method described by Smythe (cite: Ramasubramaniam, A. Phys. Rev. B 2012, 86, 115409.) and extended by Sritharan:

(cite: Berkelbach, T. C.; Hybertsen, M. S.; Reichman, D. R. Phys. Rev. B 2013, 88, 045318.)

$$V(\rho) = -\frac{e^2}{4\pi\varepsilon_0\bar{\varepsilon}}\left\{\frac{(\bar{\varepsilon}+1)(\bar{\varepsilon}-1)}{\beta_P}\left[\sum_{n=0}^{\infty}\frac{\left(\frac{\beta_N}{\beta_P}\right)^n}{\sqrt{(z-z_0-2a+2nc)^2+\rho^2}} + \sum_{n=0}^{\infty}\frac{\left(\frac{\beta_N}{\beta_P}\right)^n}{\sqrt{(z-z_0+2b+2nc)^2+\rho^2}}\right] + \frac{2(\bar{\varepsilon}-1)^2}{\beta_P}\sum_{n=0}^{\infty}\frac{\left(\frac{\beta_N}{\beta_P}\right)^n}{\sqrt{(z-z_0+2c+2nc)^2+\rho^2}} + \frac{1}{\sqrt{(z-z_0)^2+\rho^2}}\right\}$$ (Supplementary Equation 4)

where $\beta_P = (\bar{\varepsilon}+1)^2$ and $\beta_N = (\bar{\varepsilon}-1)^2$. The electron and hole are assumed to be at the center of the each MoS_2 and WS_2 monolayer along the *z* direction with a fixed distance of 6.213 Å which is the thickness of heterojunction. Then, the freedom of motion in the surface normal direction (*z*) is ignored and the excitonic quasi-particle could be simplified to two dimensions by fixing the electron-hole separation. (cite: Zhu, X.-Y., Monahan, N. R., Gong, Z., Zhu, H., Williams, K., Nelson, C. A. J. Am. Chem. Soc., 2015, 137, 8313-8320.) Furthermore, the effective mass of the excitonic quasi particle can be obtained from $\frac{1}{\mu} = \frac{1}{m_e^*} + \frac{1}{m_h^*}$, where the effective mass of monolayer MoS_2 conduction band $m_e^* = 0.506m_0$ and the effective mass of monolayer WS_2 valence band $m_h^* = 0.34m_0$, which m_0 is the free electron mass. (cite: D. Wickramaratne, F. Zahid, and R. K. Lake, J. Chem. Phys. 2014, 140, 124710.) Based on the finite element method, the SchÖringer equation, incorporated by the effective mass of the excitonic quasi particle and above Coulomb potential, have been solved on a 2×10^3 Å circle plane. The eigenvalues and selected eigenfunctions achieved from these SchÖringer equation is presented in Fig. Eigen. The *1s* CT exciton binding energy is E_{ex}= 0.44 eV, and the corresponding means radius of wave functions is $\langle\rho_{CT_{1s}}\rangle = 30.5$ Å.

Supplementary References

1. Lee, C. *et al.* Anomalous lattice vibrations of single-and few-layer MoS2. *ACS nano* **4**, 2695-2700 (2010).

2. Berkdemir, A. *et al.* Identification of individual and few layers of WS2 using Raman Spectroscopy. *Scientific reports* **3** (2013).
3. Chen, H. *et al.* Molecular Conformations of Crystalline L-Cysteine Determined with Vibrational Cross Angle Measurements. *J Phys Chem B* **117**, 15614-15624 (2013).
4. Chen, H. *et al.* Vibrational Cross-Angles in Condensed Molecules: A Structural Tool. *J Phys Chem A* **117**, 8407-8415 (2013).
5. You, Y. *et al.* Observation of biexcitons in monolayer WSe2. *Nat Phys* **11**, 477-481 (2015).
6. Rigos, A.F., Hill, H.M., Li, Y.L., Chernikov, A. & Heinz, T.F. Probing Interlayer Interactions in Transition Metal Dichalcogenide Heterostructures by Optical Spectroscopy: MoS2/WS2 and MoSe2/WSe2. *Nano Lett.* **15**, 5033-5038 (2015).
